# Endocrine Gland-Derived Vascular Endothelial Growth Factor/Prokineticin-1 in Cancer Development and Tumor Angiogenesis

**DOI:** 10.1155/2017/3232905

**Published:** 2017-03-12

**Authors:** Ana Silvia Corlan, Anca Maria Cîmpean, Adriana-Andreea Jitariu, Eugen Melnic, Marius Raica

**Affiliations:** ^1^Department of Endocrinology, “Vasile Goldis” University of Arad, Arad, Romania; ^2^Department of Microscopic Morphology/Histology, Angiogenesis Research Center, “Victor Babeș” University of Medicine and Pharmacy Timișoara, Timișoara, Romania; ^3^Department of Pathology, “Nicolae Testemitanu” University of Medicine and Pharmacy, Chișinău, Moldova

## Abstract

A lot of data suggests endocrine gland-derived vascular endothelial growth factor (EG-VEGF) to be restricted to endocrine glands and to some endocrine-dependent organs. Many evidences show that EG-VEGF stimulates angiogenesis and cell proliferation, although it is not a member of the VEGF family. At the time, a lot of data regarding the role of this growth factor in normal development are available. However, controversial results have been published in the case of pathological conditions and particularly in malignant tumors. Thus, our present paper has been focused on the role of EG-VEGF in normal tissues and various malignant tumors and their angiogenic processes.

## 1. Introduction

A great amount of data regarding the biological processes that govern cancer progression and metastasis have been gathered in the last three decades. Numerous growth factors and their receptors have been discovered and characterized. Based on these findings, the idea of a targeted therapy arose. This type of therapy was rapidly introduced in clinical practice. One of the most common drugs applied in targeted therapy is, most probably, the humanized monoclonal antibodies trastuzumab and bevacizumab.

Endocrine gland-derived vascular endothelial growth factor (EG-VEGF) was discovered and characterized in the adrenal gland 15 years ago. Since then, a lot of data suggested EG-VEGF to be restricted to endocrine glands and to some endocrine-dependent organs. Many evidences show that EG-VEGF stimulates angiogenesis and cell proliferation, although it is not a member of the VEGF family. Several data regarding the role of this growth factor in normal development are available. However, controversial results have been published in case of pathological conditions and, particularly, in malignant tumors.

In 2001, Li et al. isolated two peptides that induce smooth muscle contraction in the gastrointestinal tract of rodents; these peptides were called prokineticins [1]. In the same year, LeCouter et al. discovered a new factor, with increased levels of expression in the placenta, ovary, testis, and in the adrenal gland [2–9], and noticed that this factor is a mitogenic agent for endothelial cells in the endocrine glands. However, it seems that it did not act on brain capillary endothelial cells [8]. This agent was named EG-VEGF but it did not share sequence identity with VEGF. EG-VEGF appeared to be identical to prokineticin-1 or PROK-1 [8]. EG-VEGF is expressed predominantly in the endocrine glands and reproductive organs, and PROK-2 or Bv8 is associated with the nervous system [1, 10, 11].

## 2. The Basic Structure of EG-VEGF

EG-VEGF, also known as prokineticin-1, is a member of a new protein family. The first member of this family to be described was VPRA or venom protein A [4], also called “MIT-1” [12]. This protein was extracted from the venom of the black mamba snake, and it has been shown to be nontoxic. EG-VEGF shows a high degree of structural homology with VPRA in proportion of 80% [13]. Thus, EG-VEGF is considered to be the human orthologue of VPRA. Other members of this family include Bv8 [14], the secreted protein from the frog *Bombina variegata*, its mammalian orthologues [15, 16]. Also included herein is the digestive enzyme colipase [13] and the protein Dickkopf [3, 17, 18], which is an inhibitor of Wnt signaling. The gene that encodes EG-VEGF is located on chromosome 1p21, and the EG-VEGF protein is structurally similar to the Bv8 protein, the proportion of homology being 70–76%, and also to its murine and human orthologues.

## 3. Brief Overview of EG-VEGF: Its Expression and Functions

There are some differences between VEGF and EG-VEGF, at least as far as their temporospatial expression is concerned. For example, the expression of both VEGF and EG-VEGF has been identified in the luteal body. The mRNA for VEGF has been detected during the initial stage of the luteal body, at the same time with the formation of a capillary plexus. EG-VEGF is expressed throughout the mid- and early-late luteal stage. These observations suggest that EG-VEGF plays an important role in the persistence of the luteal body function [13].

Human placenta is well known to be particular regarding its development with special emphasis to blood vessel formation. Human placentation is closely related to the presence of EG-VEGF secreted by the syncytiotrophoblast layer with the highest expression detected between the 8th and 10th week of gestation in the normal placenta [4]. The same authors stated that, because its regulation by hypoxia and its complementary expression localisation to that of VEGF, EG-VEGF is involved in the pre-eclampsia pathogenesis. Recently published papers gave new insights into the EG-VEGF involvement in normal and pathological placental angiogenesis [12] and, maybe more important, its dysregulations impeding trophoblast proliferation and its decidual invasion [14] or pregnancy loss [15]. Also, the expression of both factors, VEGF and EG-VEGF, was identified in adrenal carcinomas. This finding supports the hypothesis, according to which the complete inhibition of angiogenesis in these tumors depends on the inhibition of both growth factors [16]. The capillaries located in the kidney and endocrine glands have fenestrated endothelial cells. The acquisition of this phenotype is highly dependent on the secretion of VEGF [19]. LeCouter et al. have shown that, in association with VEGF, EG-VEGF contributes to the regulation of angiogenesis in the human ovary [20].

Recent research suggests that prokineticins and their receptors may play a role in male reproduction due to their expression in the male reproductive organs such as the testis and the prostate [21, 22]. Its expression in the female reproductive organs, such as the ovaries, the uterus, and several tissues during pregnancy, has been widely studied [3, 4, 13]. Recently, attempts to demonstrate the implication of EG-VEGF in the pathogenesis of endometriosis have been made.

The authors Lee et al. showed an upregulation of EG-VEGF, but not of the well-known angiogenic factor VEGF, in ectopic endometriotic tissues. Also, they have demonstrated low or absent levels of expression for EG-VEGF receptors (PROKR1 and PROKR2) in ectopic endometriotic tissues. These observations imply that EG-VEGF may act as an endocrine/paracrine angiogenic factor that stimulates the formation of new blood vessels in the adjacent tissues. These data may hold importance in discovering the causes of endometriosis and possibly the medical treatment needed for its prevention [23]. A recent study also demonstrated that the expression of EG-VEGF is detectable at low levels in the normal human prostate, but its expression is markedly increased in prostate carcinoma [22]. Relatively less data are available in literature regarding the expression of prokineticin-1 and prokineticin-2 in human cancers. A recent paper showed the expression of EG-VEGF in colorectal cancer tumor cells expressing the *β*-isoform of the estrogenic receptor, being influenced by hormones [24]. The increased expression of EG-VEGF induces liver metastasis in colorectal cancer. Recent discoveries suggest that prokineticins, expressed at higher levels in cancers compared to normal tissues, may be cancer specific and, possibly, tissue specific [22]. These aspects could open new windows for cancer-specific therapies. Pasquali et al. showed a progressive increase of the expression of the EG-VEGF protein during the evolution of prostate cancers, from low-medium to high grade. These observations imply that prokineticins should be considered as prognostic biomarkers for the progression of the prostate carcinomas [22].

## 4. The Mechanism of Action of EG-VEGF Molecule

EG-VEGF (also known as prokineticin-1) and prokineticin-2 act on 2 receptors, namely, PROKR1 and PROKR2 [14, 25, 26]. Receptor activation stimulates intracellular calcium levels, the turnover of phosphoinositide and the induction of mitogen-activated protein kinase (MAPK). These signaling pathways cast some light on the actions of prokineticins on smooth muscle contraction and, particularly, on angiogenesis.

**Figure 1:**
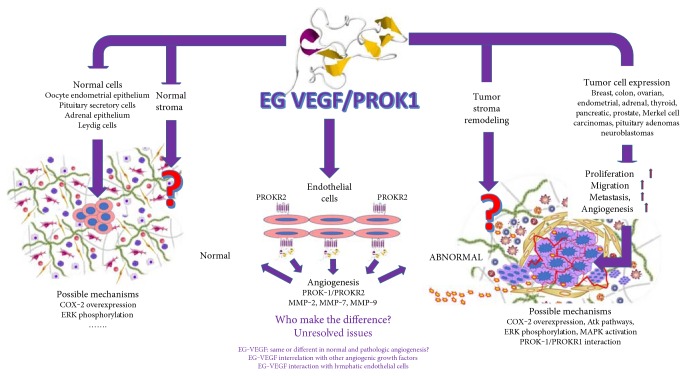
Normal tissues and malignant tumors where EG-VEGF was reported. No data about EG-VEGF interaction with normal and tumor stroma have been reported before, this being an unexplored field of EG-VEGF action, except angiogenesis (red big question mark). Other controversy should be elucidated regarding differences between EG-VEGF pathways in normal and pathologic angiogenesis.

EG-VEGF induces phosphorylation of the mitogen-activated protein kinases, ERK1 and 2, and the Akt serine/threonine kinase of the phosphatidylinositol 3 kinase cell survival pathway [27]. It seems that EG-VEGF activates both pathways in both types of endothelial cells in the human placenta [28] These data support the hypothesis according to which EG-VEGF is a new growth factor that stimulates proliferation and survival of endothelial cells in the human placenta [28]. Also, interaction between EG-VEGF and its corresponding receptors induces inositol phosphate mobilization and sequential phosphorylation of c-Src, ERK1, and epidermal growth factor receptor [29]. Additional pathways of EG-VEGF action to exert an angiogenic role may be related to its proinflammatory function mediated by its ability of time-dependent increase in the expression of IL-8 and COX-2 [30]. Several data reported that EG-VEGF involvement in tumor angiogenesis has a specific organ-dependent pathway. In pancreatic cancer, new blood vessel development is induced and sustained by EG-VEGF secreted by pancreatic islet cells and stellate cells, as a result of its interaction with TGF-*β*1 and PDGF-A [31]. In adrenal tumors, EG-VEGF interacts with steroidogenic factor 1 (SF-1) inducing a nuclear expression of EG-VEGF which proved to have a strong impact on prognosis for patients with adrenal tumors [32]. One of the most well-characterized malignancies in relation to EG-VEGF expression is colon cancer. Tabata et al. [33] reported EG-VEGF as a factor to strengthen cell invasion ability in colon cancer cell lines, by acting on MMP-2, MMP-7, and MMP-9 via prokineticin receptor 2. Later, Goi and his team defined EG-VEGF and its corresponding receptor PROKR2 as prognostic markers in colorectal carcinoma [34, 35] suggesting a common pathway with VEGF sustained by their finding regarding the enhancement of antitumor effects when it was simultaneously targeting VEGF and EG-VEGF [36]. Fewer data were reported in normal tissues about EG-VEGF mechanism of action. Most of these data are related to human reproduction. Eddie et al. described the action of EG-VEGF being highest in the midgestation (17–21 weeks) ovaries by the increase of ERK phosphorylation and elevated COX-2 expression [37]. It seems that EG-VEGF levels in normal ovaries influence the endometrium receptivity during implantation. High-quality oocytes obtained during in vitro fertilization stimulation had a high vascularity given by high levels of EG-VEGF in both follicular fluid and serum of women who followed such procedure. Cases with well-vascularized oocytes had also a well-vascularized peri-implantation endometrium favoring the increase of clinical pregnancy rate and embryo maturation [38]. General mechanisms and unresolved issues regarding EG-VEGF involvement in normal and pathologic processes are summarized in Figure 1.

## 5. EG-VEGF Expression in Normal Tissues

EG-VEGF has been identified in the ovary, placenta, testis, and in the adrenal glands [20, 29]. The name “EG-VEGF” is based on its action on the capillary endothelial cells in the endocrine glands [20]. It has been demonstrated that EG-VEGF stimulates proliferation, chemotaxis, and survival of endothelial cells from steroidogenic tissues. As it has already been mentioned, EG-VEGF, also called prokineticin-1, is part of a family of proteins that includes Bv8 or prokineticin-2. Both agents act on 2 types of receptors, known in scientific literature as PROKR1 and PROKR2 [27]. The expression of EG-VEGF and its receptors is increased in the human placenta during the first trimester of pregnancy. The highest level of expression was evidenced in the syncytiotrophoblast layer. It has also been shown that this factor controls trophoblast invasion. The expression of EG-VEGF and its receptors is increased not only in normal conditions but also in pathological situations such as hypoxia. It appears that the plasma levels of EG-VEGF are higher in pregnant women diagnosed with pre-eclampsia [4, 5, 28]. The placenta is known to hold the highest vessel density in the human organism. Numerous studies regarding the proangiogenic action of VEGF have used placenta specimens. It seems that VEGF has different effects on the 2 types of endothelial cells identified at this level. One of these cell types is the human placental microvascular endothelial cells (HPECs), located in the fetal capillaries of the chorionic villi. The other cell type is represented by the human umbilical vein macrovascular endothelial cells (HUVECs).

## 6. EG-VEGF-Induced Angiogenesis

Numerous research papers have been focused on the proangiogenic role of EG-VEGF.

LeCouter et al. did not demonstrate a proliferative effect of EG-VEGF on the endothelial cells from the human umbilical vein [20]. Brouillet et al. confirmed the data shown by Ferrara et al. [39].

It appears that EG-VEGF has no effect on the proliferation and migration of endothelial cells from the human umbilical vein. However, EG-VEGF plays an important role in placental angiogenesis, which is reflected by its selective action on human placental microvascular endothelial cells. These endothelial cells belong to the fetal capillaries of chorionic villi [39]. Endothelial cells of the human umbilical vein have been used in many scientific papers as a model for endothelial cells [40, 41]. These endothelial cells are macrovascular endothelial cells, in contact with oxygenated blood. Microvascular endothelial cells are different from macrovascular endothelial cells as far as gene expression, phenotype, and physiology are concerned [42, 43].

The difference between EG-VEGF action on the microvascular and macrovascular endothelial cells of the placenta could be explained through the different levels of intracellular Gαi1 and Gαi2 protein expression. In microvascular endothelial cells, the expression of G*α*i2 was 3 times higher and the expression of G*α*i1 was 3 times lower, in comparison to the macrovascular endothelial cells. These data are identical to those shown by Masri et al. [44]. Brouillet et al. [39] showed that Gi*α*2-positive cells present a higher degree of inhibition of the enzyme adenylate cyclase in comparison to cells that mainly express Gi*α*1 [44, 45].

Several studies demonstrated an increased expression of EG-VEGF receptors, PROKR1 and PROKR2, in the placenta during the first trimester of pregnancy [4, 5, 28].

These data are confirmed by Brouillet et al. [39]. EG-VEGF receptors are predominantly expressed in microvascular endothelial cells compared to macrovascular endothelial cells. This could explain the effects of EG-VEGF on microvascular endothelial cells. The differential effects mediated by both receptors were investigated by means of siRNA and blocking antibodies in the microvascular endothelial cells. It has been concluded that PROKR1 mediates the angiogenic effects of EG-VEGF and that PROKR2 mediates EG-VEGF action on cell permeability. The same actions of the 2 receptors have also been demonstrated in cardiomyocytes [46–48].

Considering the 87% structural homology in the amino acid sequence of PROKR1 and PROKR2, their differential action could be partially explained through the use of different signaling pathways. Also, the recruitment of the G protein group by these receptors may result in differential effects. Slessareva et al. demonstrate that coupling selectivity depends on the intracellular concentration of G proteins [49].

It has been shown that EG-VEGF stimulates maternofetal exchanges through its action on microvascular endothelial cells' permeability [39]. The placental capillaries are not fenestrated, and the endothelial cells are interconnected by adherent and tight junctions [50–52].

Recent data show that hepatic sinusoidal cells exclusively express PROKR2. PROKR2 induces the internalization of ZO-1, a structural protein of junctional complexes and intercellular adhesions [48]. It may thus be possible that EG-VEGF action on vascular permeability implies the regulation of tight junction proteins.

The authors [39] support the hypothesis that EG-VEGF controls the process of angiogenesis during the first trimester of pregnancy and at term.

### 6.1. EG-VEGF Plays a Significant Role in the Development of the Placental Vasculature

Human placentas at term are characterized by an increased angiogenesis in order to allow a high blood distribution. Scientific data point out that EG-VEGF stimulates vascular organization, permeability, and sprouting of the placental microvascular endothelial cells in a similar manner to the well-characterized growth factors VEGF and FGF-2.

Vural et al. [38] showed that EG-VEGF-induced angiogenesis in the ovary is similar to that induced by VEGF. Recently, it has been evidenced that EG-VEGF, expressed and secreted from the syncytiotrophoblasts, exerts its action on the extravillous trophoblast cells. This aspect supports the important role of EG-VEGF in human placental angiogenesis [39].

Inhibitors of the growth factor EG-VEGF could improve our understanding of the role of EG-VEGF in normal and tumor angiogenesis. Our team attempted to study the impact of an anti-EG-VEGF antibody on the chick embryo chorioallantoic membrane [53]. The study made use of the chick embryo chorioallantoic membrane as an experimental model because of its similarities to the human placenta. Through the use of anti-EG-VEGF antibodies, different changes in the main vessels and capillaries of the chick embryo chorioallantoic membrane were observed. The main vessels suffered vasodilatation without any compromise noticed in their structure. The major changes were observed in the capillaries, namely, the breakage of endothelial cells and intimal discontinuities. These changes allowed an increased bleeding into the chorion. Moreover, the treatment with anti-EG-VEGF antibodies reduced the proliferative potential of the endothelial cells. The authors concluded the possibility of an avian type of EG-VEGF, with an important role in the development of the chorioallantoic membrane and embryonic vasculature.

## 7. The Expression of EG-VEGF in Tumor Cells and Its Relationships with the Patients' Prognosis

Recent studies showed overexpression of prokineticin-1 not only in several types of cancers such as colorectal, pancreatic, and prostatic carcinoma but also in carcinomas of the testis and in neuroblastomas [24, 54].

Interestingly, mRNA EG-VEGF is not expressed in the normal colorectal mucosa, but EG-VEGF was detected in all colorectal cancer cell lines [24]. Estradiol, together with the selective estrogen receptor modulator tamoxifen, influences the proliferation rate and the intracellular signal transduction in colorectal cancer [55].

Arai et al. [56] identified the expression of estrogen receptor *β* in colorectal cancer cell lines, thus establishing a link between colorectal cancer and hormone influences.

Japanese authors analyzed the association between EG-VEGF and colorectal cancer and identified the expression of this growth factor in 5 out of the 6 tumor cell lines [24].

EG-VEGF plays an important role in tumor angiogenesis, causing an exponential increase of the tumor. Folkman observed an amplification of the tumor volume caused by the formation of vascular networks during the process of angiogenesis [57]. Kim et al. [58] reported an active angiogenesis in tumors associated with a high proliferation rate.

EG-VEGF-stimulated angiogenesis induces cell proliferation, a high microvessel density being correlated with an increased proliferation index.

The experiment carried out by the Japanese authors [24] consisted in the transfection of colon cancer cell lines with the gene expressing EG-VEGF. During in vivo examination, the cell proliferation rate in subcutaneous implants and in the cecum implanted with cells was markedly increased in colorectal cell lines transfected with EG-VEGF. The techniques used in order to analyze the relationship between EG-VEGF and angiogenesis were dorsal air sac analysis and immunohistochemistry. In colon cancer cell lines that were transfected with EG-VEGF gene, the microvascular count increased [24].

### 7.1. EG-VEGF Clearly Stimulates the Proliferation of Tumor Cells and Metastases

The occurrence of metastases after the implantation of colon cancer cell lines, transfected with EG-VEGF, was experimentally analyzed in the spleen of laboratory mice. Metastases were observed in the liver. Treatment of the colorectal cancer cell lines that overexpressed EG-VEGF, with antisense EG-VEGF oligonucleotides that were subcutaneously injected into mice, produced the inhibition of angiogenesis and tumor growth consecutively [24].

Other scientific papers evaluated the role of EG-VEGF-induced pathological angiogenesis and tumor growth. One of the main causes of death in gynecological diseases is represented by ovarian cancers. Several studies show VEGF overexpression in ovarian cancer. VEGF plays a pivotal role not only in the process of angiogenesis but also in the neoplastic transformation of the epithelium that covers the surface of the ovary [59].

From this point of view, attempts to block VEGF action in the advanced stages of ovarian carcinomas seem legit. However, some clinical trials that made use of anti-VEGF monoclonal antibodies did not obtain the expected benefits [60]. The explanation for these unexpected results could partially be determined by the complexity of the angiogenic process. Angiogenesis is orchestrated by a great number of growth factors such as PDGF or FGF. These molecules induce the proliferation of endothelial cells and tumor growth [61, 62].

The role of EG-VEGF in the process of angiogenesis associated with ovarian carcinomas was less studied [63]. The authors attempted to find a correlation between EG-VEGF expression and prognosis in epithelial ovarian carcinomas.

### 7.2. The Role of EG-VEGF in Tumor Angiogenesis: Direct Proportionality between EG-VEGF Expression and Microvascular Density

EG-VEGF, a novel organ-specific proangiogenic molecule, is expressed in the ovary, the testis, the adrenal cortex, and especially in the placenta. Low levels of mRNA EG-VEGF were detected in the colon, the small intestine, the liver, spleen, brain, thymus, and, recently, in the anterior pituitary gland [64].

EG-VEGF expression was evaluated in different types of cancers such as colorectal cancer [24], ovarian carcinoma [65], and pancreatic adenocarcinoma [66]. EG-VEGF was also detected in polycystic ovary syndrome [13].

As previously stated, EG-VEGF possesses an important role in normal and pathological angiogenesis [66]. Recently, it was confirmed that this growth factor regulates the growth and survival of tumor cells [67].

The involvement of EG-VEGF in the process of physiologic and pathological angiogenesis in the human ovary is confirmed by current data [68].

By means of RT-PCR, Zhang et al. [65] did not detect EG-VEGF expression in ovarian cancer cell lines or cultured human ovarian surface epithelium. Surprisingly, Bălu et al. showed a positive reaction for EG-VEGF in the epithelial cells of the ovarian surface [63]. Overexpression of EG-VEGF in ovarian cancers that arise from the surface epithelium could thus be taken into consideration.

Fraser et al. [3] point out that the expression of EG-VEGF in the human luteal body undergoes cyclic variations.

An increased percentage of ovarian tumors are positive for EG-VEGF, showing 3 different patterns of expression [63]. There is a certain degree of specificity in the EG-VEGF expression pattern for various histopathologic types of ovarian carcinomas. The distribution of EG-VEGF-positive tumor cells located in the tumor mass and in the stroma suggests the possibility that EG-VEGF is implicated in the progression and metastasis of ovarian tumor cells. Similar findings were reported by Nagano et al. in colorectal carcinoma [69].

Besides EG-VEGF, other members of the heparin-binding growth factor family are expressed in ovarian cancer. One of these heparin-binding growth factors is heparin-binding EGF-like growth factor. EGF-like growth factor appears to interact with EGFR, thus activating downstream signaling pathways. These signalizing pathways are responsible for the acquisition of a malignant phenotype that includes metastasis and resistance to treatment [70]. All cases of advanced ovarian carcinoma (stages III and IV) were characterized by the presence of EG-VEGF-positive cells, located at the periphery of the tumor [63]. EG-VEGF may thus be implicated in determining the invasive behavioral phenotype of the tumor [63].

Despite these findings, Zhang et al. reported the expression of EG-VEGF in early stages of ovarian carcinoma. EG-VEGF expression was decreased in advanced stage ovarian carcinoma [65].

Another study led by Ngan et al. identified the expression of EG-VEGF in the normal peri-implantation endometrial tissue specimens from women during their reproductive period. mRNA EG-VEGF levels were rarely reported in endometrial tissue specimens from postmenopausal women and women with endometrial carcinoma [71].

The role of EG-VEGF in angiogenesis associated with prostate carcinoma has been studied by a group of Italian scientists [22]. It is well known that angiogenesis plays a fundamental role in the growth, invasion, and metastasis of prostate cancer [72, 73]. Prostate cells can synthesize and secrete growth factors that orchestrate the process of angiogenesis. One of these factors is VEGF. In most prostate cancers, an increased expression of VEGF and a high microvascular density, respectively, have been identified. These results support the role of angiogenesis in the growth of prostatic carcinoma. Increased levels of VEGF were correlated with a poor prognosis [73–77].

A decreased level of EG-VEGF mRNA expression was observed in the normal epithelial prostate cells. Its expression increases in human prostatic carcinoma, directly proportional with the advance of the Gleason score. EG-VEGF represents an important prognostic marker for the progression of prostatic carcinoma.

Previous data report that EG-VEGF mRNA levels are barely identified by Northern blot in the normal human prostate [20]. EG-VEGF expression was evaluated in the normal prostate tissue, in benign prostate hyperplasia, and in prostate cancer cell lines [22]. The normal human prostate is characterized by low levels of EG-VEGF. EG-VEGF expression appears to be increased in prostate carcinoma. Western blotting and immunohistochemistry were used in order to detect EG-VEGF mRNA and protein levels in prostate epithelial cells. EG-VEGF expression was amplified as the Gleason score advanced. PROKR1 and PROKR2 transcripts were identified in epithelial cell cultures of the normal and malignant prostate. These results suggest that EG-VEGF interacts with the 2 receptors located on the surface of prostatic epithelial cells. Pathological angiogenesis has been linked to pathways that control apoptosis, to the effects of androgen receptor, and to different cytokines and cell adhesion molecules [78–82]. EG-VEGF could play an important role in the process of angiogenesis associated with prostate carcinoma [22]. Prostate carcinoma specimens were studied by using 3 established cell lines, namely, PC3, DU-145, and LNCaP, as cell culture models [83].

Comparative studies carried on human prostate carcinomas are regarded as being the most useful. These studies are based on donor-matched pairs of normal and malignant primary cultures [84]. Sinisi et al. studied the expression of EG-VEGF and its 2 receptors in primary cultures of normal (NPEC) and malignant (CPEC) epithelial prostate cells. Also, EPN, a nontransformed human prostate epithelial cell line, was included in the study [85]. A higher level of EG-VEGF expression was observed in prostatic carcinoma specimens in comparison to the normal prostatic tissue. EG-VEGF may thus be considered a cancer-specific molecule. A decreased level of EG-VEGF mRNA expression was observed in the normal epithelial prostate cells (NPEC) and in the EPN cell line. EG-VEGF protein appeared to be absent in the normal prostate. These observations could suggest that EG-VEGF is inactivated in normal tissues or that its signaling pathway is inhibited.

The acquisition of a malignant phenotype is associated with the progressive activation of EG-VEGF protein expression. This aspect could be explained by the fact that EG-VEGF plays an important role in tumor angiogenesis. It induces endothelial cell proliferation, migration, and fenestration and stimulates tumor growth consecutively.

EG-VEGF could represent an important prognostic marker for the progression of prostatic carcinoma [22]. As prostate carcinomas progress from a low-medium grade to a high grade, a concordant progressive increase was observed in the EG-VEGF protein level [22].

Literature data show the key role of angiogenetic factors in the testis. Microvessel remodeling is described not only during the normal development of the testis but also in pathologic conditions such as testicular cancer [86–89].

The discovery of the role of EG-VEGF in testicular cancer angiogenesis seems promising due to the fact that the incidence of these cancers has lately increased. Testicular carcinoma has become the most frequent malignancy in 25- to 35-year-old men [90, 91].

VEGF expression was identified in different histopathological types of testicular cancer, especially teratomas [88].

During fetal development, EG-VEGF induces angiogenesis which is essential for the transport of testosterone, synthesized in Leydig cells. Testosterone is transported to other target tissues. Also, angiogenesis is needed in order to ensure the access of gonadotropins and other regulatory proteins from the periphery to the testis.

A group of researchers from Denmark attempted to evaluate the expression of EG-VEGF, mRNA, and protein, in normal fetal and adult testes and also in different cell types of testicular cancers [92]. The authors used monoclonal antibodies against EG-VEGF protein and concluded that its expression in normal adult testis was restricted to the Leydig cells [92]. The immunohistochemical observations confirmed those obtained by in situ hybridization methods [9]. The restriction of EG-VEGF expression in the Leydig cells differs from that of VEGF, which was evidenced in both Leydig and Sertoli cells [9]. The authors observed EG-VEGF expression in the Leydig cells of the human fetal testis, starting from week 14 of gestation. Interestingly, the fetal production of STAR (steroidogenic acute regulatory protein) also starts during week 14 of gestation. This protein regulates testosterone production by Leydig cells [93, 94]. The fact that the expression of EG-VEGF also begins at week 14 of gestation suggests that this growth factor may be crucial for the normal endocrine function of the testis [92, 95].

Research studies point out the existence of a binding site for steroidogenic acute regulatory protein in the promoter region of the EG-VEGF gene. This fact implies that hormones regulate the expression of EG-VEGF. There are several candidates for the hormonal regulation of EG-VEGF expression and secretion. The best candidate would be LH, which modulates the genes involved in testosterone synthesis [96–98]. However, ACTH was recently shown to stimulate the synthesis of testosterone in fetal testis [99].

The expression of EG-VEGF in the testis is restricted to the Leydig cells. EG-VEGF, and not VEGF, plays the pivotal role in the process of angiogenesis that occurs in Leydig cell tumors. EG-VEGF thus promotes the growth of the Leydig cell tumors.

The expression of EG-VEGF, both at mRNA and protein level, was restricted to the Leydig cell tumors [80]. Its expression was not detected in germinal cell tumors or Sertoli cell tumors. In samples associated with CIS (carcinoma in situ), an increased expression of EG-VEGF was identified, in comparison to normal tissue. In situ hybridization and immunohistochemistry were used in order to show that the Leydig cells located around the tubules containing CIS actually expressed EG-VEGF.

Some literature reports show that STAR is expressed predominantly in Leydig cell tumors [92]. It is also known that Leydig cell tumors synthesize and secrete testosterone and are controlled by LH [100, 101].

The data regarding the correlation established between VEGF and microvessel density (MVD) in testicular cancers are scarce [90, 102]. VEGF is expressed in seminomas and nonseminoma germinal cell tumors [88].

Microvessel count was clearly higher in Leydig cell tumors compared to seminomas [92]. VEGF expression, however, was not remarkable in Leydig cell tumors.

Very few articles have focused on the expression of EG-VEGF in the normal human hypophysis and only one scientific paper reported its presence in pituitary adenomas [103].

**Figure 2:**
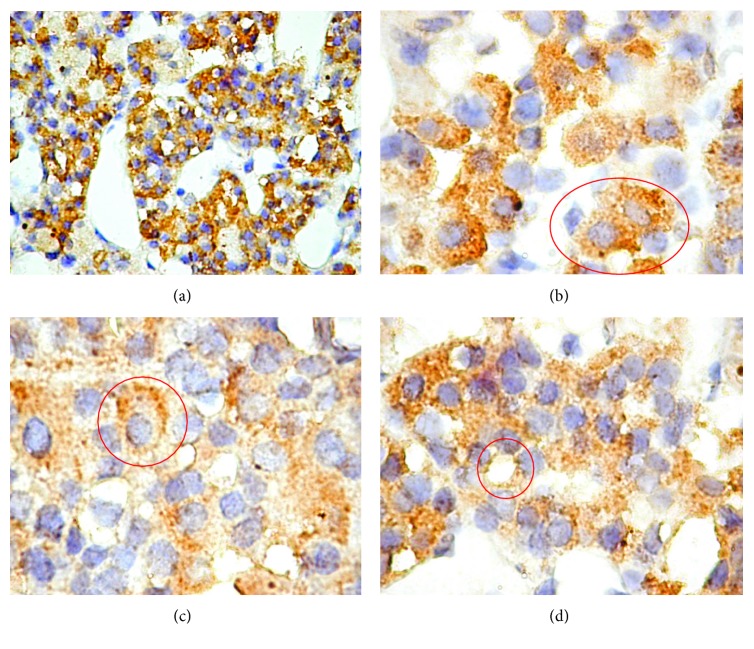
EG-VEGF expression in normal pituitary gland with a heterogeneous granular pattern (a). Acidophilic cells have a homogeneous (b) or heterogeneous (c) granular cytoplasmic expression of EG-VEGF, while chromophobe cells had a weak granular pattern of EG-VEGF expression (d).

The research carried by Raica et al. revealed the differential expression of EG-VEGF in the normal pituitary gland (Figure 2(a)). The acidophilic cells located in the anterior pituitary gland (Figure 2(b) and Figure 2(c)) and the chromophobe cells (Figure 2(d)) were strongly positive for EG-VEGF. EG-VEGF expression was not detected in basophilic cells. The authors wished to extend the study regarding the role of EG-VEGF in the development and progression of human pituitary adenomas.

Previous research articles evidenced a downregulation of EG-VEGF expression in human pituitary adenomas in comparison to the normal pituitary tissue specimens. The abovementioned research study resulted in data that are in contradiction with previous results. The cases used in the study showed an overexpression of EG-VEGF in over 50% of the pituitary adenoma cases. The acidophilic solid pattern and acidophilic or basophilic papillary pattern were predominantly observed.

Compact pituitary adenomas with acidophilic cells present an increased expression of EG-VEGF correlated with an overexpression of EGFR. Through the activation of this receptor, EG-VEGF inhibits apoptosis of adenoma cells.

Compact pituitary adenomas with acidophilic cells have shown an overexpression of EG-VEGF. A significant correlation was found between EG-VEGF and EGFR overexpressions. The interaction between EG-VEGF and EGFR has been already mentioned in the placental changes responsible for pregnancy loss [64, 104]. EG-VEGF induces EGFR phosphorylation and consequently its activation [104]. EG-VEGF thus possesses an antiapoptotic role through promoting the survival of tumor cells. The study entitled “The expression and prognostic significance of EG-VEGF in pituitary adenomas” demonstrated for the first time the correlation between EG-VEGF and EGFR in pituitary adenomas such as the compact acidophilic pituitary adenomas. Based on these results, EG-VEGF expression in compact acidophilic pituitary adenomas was not influenced by the hormonal profile.

**Figure 3:**
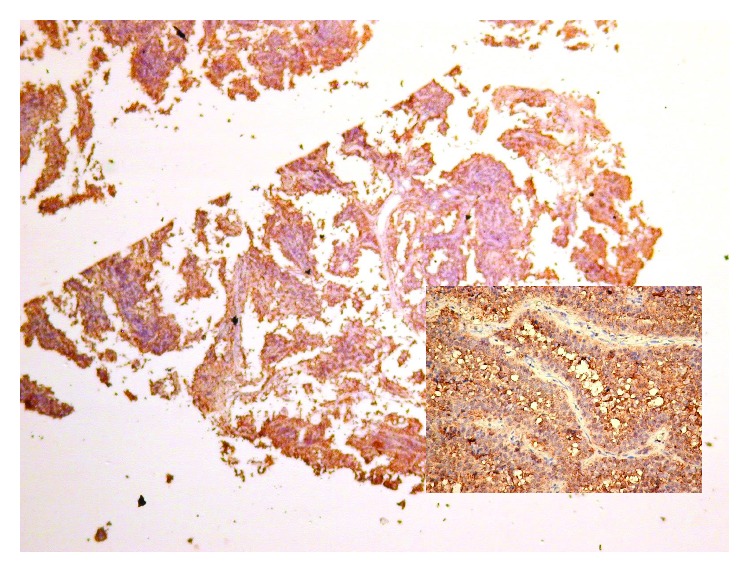
Pituitary adenoma with papillary morphology, with high expression of EG-VEGF.

Papillary-type pituitary adenomas are characterized by an increased expression of EG-VEGF (Figure 3), that is correlated with an increased expression of prolactin. Both EG-VEGF and prolactin induce an increased proliferation rate of adenoma cells.

The lack of correlation between EG-VEGF and EGFR in papillary-type pituitary adenomas could be explained through the different molecular profiles of pituitary adenomas. In papillary-type pituitary adenomas, the expression of EG-VEGF correlated with the expression of prolactin.

Data that refer to the interaction between EG-VEGF and the plasma level of prolactin have been published in literature with regard to the differential diagnosis of hypogonadism [30]. The decrease in the plasma levels of prolactin was caused by genetic mutations in prokineticins. Both, EG-VEGF and prolactin, are recognized as growth factors that induce cell proliferation.

Pituitary adenomas with acidophilic cells, most of which immunostained for prolactin, presented an overexpression of EG-VEGF. At the same time, they were characterized by an increased proliferation rate that significantly correlated with the expression of EG-VEGF. It has been concluded that EG-VEGF and prolactin have a synergistic effect on the proliferation rate of tumor cells and that it is involved in tumor growth.

As opposed to the basophilic cells of the normal pituitary gland, that were negative for EG-VEGF, the basophilic cells of pituitary adenomas had a relatively increased expression of EG-VEGF. The only growth pattern that was identified in case of pituitary adenomas with EG-VEGF-positive basophilic cells was the papillary pattern. Literature data support the involvement of EG-VEGF in the determination of the papillary growth pattern of tumors arising from endocrine glands [105] such as the thyroid. These types of thyroid carcinomas show BRAF mutation, which induces an aggressive behavior, such as the tumors' infiltrative capacity and the occurrence of lymph node metastases [105].

In pituitary adenomas with basophilic cells, EG-VEGF overexpression correlates with the expression of PDGF-A and, especially, with that of PDGF-B.

Pituitary adenomas with basophilic cells that expressed EG-VEGF were also positive for PDGF-A and PDGF-B. The correlation between these 3 growth factors was statistically significant and stronger for PDGF-B. PDGF-A and PDGF-B were also studied and confirmed to be involved in the pathogenesis of papillary-type thyroid carcinomas [106–108] that are associated with an unfavorable prognosis.

In agreement with other studies [103], a statistically significant correlation was found between the overexpression of EG-VEGF and the expression of LH. A scientific paper that describes the correlation between these 2 markers in pituitary adenomas is available in literature. Other studies that were focused on their association attempted to identify biomarkers for fertility and implantation [109, 110].

The significant correlation between the expression of EG-VEGF and the expression of GFAP and S100 protein could imply the action of this growth factor on follicular-stellate cells from the normal human pituitary gland and from the pituitary adenomas.

The statistically significant correlation found between the expression of EG-VEGF and the expression of GFAP and S100 protein suggests a possible role of EG-VEGF in the reactivity of follicular-stellate cells in pituitary adenomas. There are no data that confirm the synthesis of EG-VEGF in follicular-stellate cells from pituitary adenomas. Also, there is no information regarding the action of EG-VEGF on follicular-stellate cells from the normal human pituitary gland and from pituitary adenomas.

## 8. Future Perspectives

VEGF has long been recognized as fundamental factor in the process of physiologic and pathologic angiogenesis. From this point of view, the use of VEGF pathway inhibitions seems legit. However, anti-VEGF antibody-based therapy is known to have side effects such as the induction of endothelial cell apoptosis and the interference with vasculature functionality. The identification of EG-VEGF gene by LeCouter et al. may initiate the discovery of tissue-specific antiangiogenic molecules and consequently a reduction of these systemic side effects.

The discovery of EG-VEGF may pave the way for the development of angiogenesis inhibitors that possess specificity for some types of cancer. Recently published data strongly suggested the crucial role of EG-VEGF into the human reproduction pathology with a special emphasis to implantation process related to infertility (see Table 1) [111, 112]. Despite several studies developed in the field of carcinogenesis and angiogenesis regarding EG-VEGF-related pathways, a lot of malignant diseases need further investigation for elucidating EG-VEGF role in their pathogenesis (Table 1) [113–116].

## Figures and Tables

**Table 1:** Most recent papers reporting EG-VEGF involvement in human reproduction pathology and malignancies where EG-VEGF's role is less elucidated.

Authors	Year	Pathology	Brief overview of EG-VEGF role	Biomarker	Ref.
Wang et al.	2016	Implantation, trophoblast invasion, and ciliogenesis	Interrelation between EG-VEGF, ERK1/2 activation, and intraflagellar transporter required for ciliogenesis IFT88	No	[111]
Morales et al.	2016	Breast cancer	EG-VEGF heterogeneity expression with no impact on diagnosis and prognosis in breast cancer	No	[113]
Jayasena et al.	2016	Miscarriage	Serum level of EG-VEGF failed to be associated with miscarriage	Not validated as serum biomarker	[112]
Li et al.	2010	Multiple myeloma	Multiple signaling pathway activation, Mcl1 upregulation, proliferation, and survival of multiple myeloma cells	No	[114]
Nakazawa et al.	2015	Sporadic colorectal cancer	Significantly higher in cases with serosal invasion, lymphatic invasion, venous invasion, lymph node metastasis, liver metastasis, hematogenous metastasis, and higher stage disease	Potential biomarker for worse prognosis, invasion, and metastasis	[115]
Li et al.	2006	Human hepatocellular carcinoma	Portal vein tumor thrombus formation promoted by angiogenesis via EG-VEGF	No	[116]
